# Ramp lesion in anterior cruciate ligament injury: a review of the anatomy, biomechanics, epidemiology, and diagnosis

**DOI:** 10.1186/s43019-023-00197-z

**Published:** 2023-08-25

**Authors:** Bo Seung Bae, Sunin Yoo, Sang Hak Lee

**Affiliations:** 1 Department of Orthopedic Surgery, Center for Joint Diseases and Rheumatism, Kyung Hee University Hospital at Gangdong, 892 Dongnam-ro, Gangdong-gu, 134-727 Seoul, Republic of Korea; 2 Department of Orthopedic Surgery, Kyung Hee University Hospital, Seoul, Republic of Korea

**Keywords:** Ramp lesion, Medial meniscus, Anterior cruciate ligament

## Abstract

Ramp lesions, commonly observed in patients with anterior cruciate ligament (ACL) injuries, have been previously defined as longitudinal tears around the meniscocapsular junction. However, the definitions and interpretations of ramp lesions have varied, emphasizing the need to confirm their presence before surgery and the importance of direct visualization using arthroscopy. Recent histological studies have reported new findings on ramp lesions, shedding light on their attachment mechanisms. The anatomical structures around the ramp lesion, such as the posterior horn of medial meniscus (PHMM), semimembranosus (SM), posteromedial (PM) capsule, and meniscotibial ligament (MTL), were assessed regarding how these structures could be attached to each other. The studies of ramp lesions have also contributed to the progression of biomechanical studies explaining the cause and effects of ramp lesions. Ramp lesion has been proven to stabilize the anteroposterior (AP) instability of ACL. In addition, various laboratory studies have demonstrated the relationship between rotational instability of the knee joint and ramp lesions. The analysis of risk factors of ramp lesion helped to understand the injury mechanism of the lesion. Many authors have evaluated the prevalence of ramp lesions in patients with ACL injuries. The development of arthroscopy techniques has influenced the outcomes of ACL reconstruction with the easy detection of ramp lesions. This review article aims to analyze the past findings and recent advancements in anatomical, biomechanical, and epidemiological studies of ramp lesions in patients who underwent ACL reconstruction, and provide various perspectives ramp lesions in patients with ACL reconstruction.

## Background

Meniscocapsular lesions of the posterior horn of the medial meniscus (PHMM) are typically related to anterior cruciate ligament (ACL) injuries [1]. These lesions were first introduced as “ramp lesions” by Strobel in 1988 as 2.5-cm peripheral longitudinal tears at the meniscocapsular junction in knees with an ACL deficiency [2]. The peripheral longitudinal tear around the meniscocapsular junction area appears to have a ramp-like inclination, with the anterior aspect of the tear located more superiorly than the posterior aspect of the tear. Ramp lesions are described by most authors as involving peripheral meniscocapsular attachment of PHMM and meniscotibial ligament (MTL) disruption [2–5]. Some authors described the lesion by adding tear length to the above concept, ranging from 0.5 to 2.5 cm [2–4, 6, 7]. A recent study shows that ramp lesions can be classified into five subtypes based on tear characteristics, locations, and stability [4]. Furthermore, the tear zone located in the red–red zone around the meniscosynovial or meniscocapsular junction [3, 4, 8] or in the red–white zone within 5 mm of the meniscocapsular junction of PHMM [9, 10] has been added to the definition of a ramp lesion.

Several case studies of ACL reconstruction have revealed that prolonged long delay in ACL reconstruction increases the incidence of medial meniscal tears [11, 12]. The prevalence of ramp lesions in patients with ACL injuries ranges from 9% to 40% [1, 3]. The prevalence reports vary because ramp lesions can be difficult to detect even with probing of the PHMM during standard anterior exploration. These lesions, therefore, are also called “hidden lesions” [4, 13, 14]. Numerous studies have found it difficult to identify these lesions with magnetic resonance imaging (MRI) owing to its moderate sensitivity, resulting in false negative errors [15]. The incidence of ramp lesions might be underestimated in previous studies.

Close examination of the preoperative MRI, including signal intensity, marginal irregularity, bone contusion around the peripheral area of PHMM, and morphometrics of the knee joint, is crucial to accurately diagnose ramp lesions in patients with acute and chronic ACL injuries. Unhealed ramp lesions can increase the knee joint’s anteroposterior (AP) and rotational instability, further exacerbating existing meniscal tears and increasing the strain on the reconstructed ACL graft [16]. This review aims to provide a comprehensive understanding and diagnosis of ramp lesions from anatomical, biomechanical, and epidemiological aspects.

## Anatomy

The medial meniscus (MM) has a semicircular shape and covers approximately 50–60% of the medial tibial plateau (MTP) [17, 18]. The width of the PHMM, which has a mean length of 11–12.6 mm, becomes gradually smaller toward the anterior horn of the MM, which has a mean width of 7.6 mm [6, 17–19]. The periphery of the body of the MM is thick and gradually becomes thinner toward the central area [19]. The anatomical structure of the MM is freely mobile at the central area and firmly attached to the peripheral area of the body in the MM to the capsule of the knee joint. It has many advantages, including shock absorption, stress reduction, joint lubrication, nutrition supplementation, and stabilization of the knee joint [19–21]. The femoral and tibial attachments of the MM make the MM less mobile than the lateral meniscus (LM), contributing to the kinematics of the knee joint and influencing various meniscal tear patterns [18]. It is necessary to analyze the detailed structures adjacent to the attachments of the MM, especially the PHMM, to understand the functions and effects of ramp lesions in patients with ACL injuries.

**Fig. 1:**
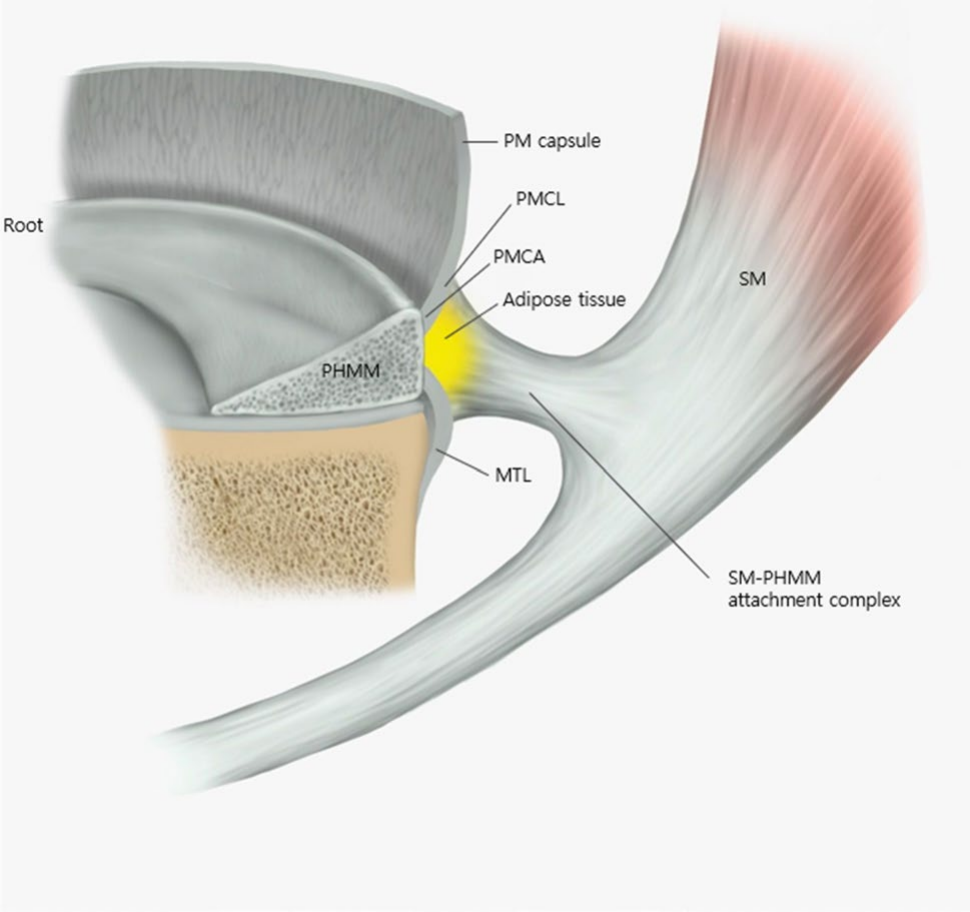
Peripheral attachments of PHMM. *MTL* meniscotibial ligament, *PHMM* posterior horn of medial meniscus, *PM* posteromedial, *PMCA* posterior meniscocapsular attachment, *PMCL* posteromedial meniscocapsular ligament, *SM* semimembranosus muscle

There are various anatomical analyses of the meniscocapsular attachment and the relationship between the posteromedial (PM) capsule and the superior edge of the PHMM (Fig. 1). Recently, Cavaignac et al. [22] reported on the meniscocapsular ligament, which connected the PM capsule to the superior edge of the PHMM in all 14 dissected cadaveric knees. The MTL consists of parallel collagen fibers histologically attached proximally to the inferior edge of the PHMM and distally to the proximal MTP. This study explains the anatomical structure and the classification of ramp lesions previously introduced by Thaunat et al. [4]. On the basis of the location of the meniscocapsular ligament and MTL around the PHMM and the fat pad signal between them on MRI, the peripheral attachments of PHMM described by Cavaignac et al. [22] are consistent with the ramp lesions observed on MRI. However, some authors have reported that the superior edge of the PHMM was not attached to any structure [6, 17, 23]. According to Dephillipo et al. [6], the PM capsule coursed distally and was connected inferiorly to the superior edge of the PHMM in all 14 fresh-frozen cadaveric knees. The attachment of the PM capsule, which was located within 36.4% of the total PHMM height, meets directly with the attachments of the MTL at the posterior site of the meniscocapsular junction, forming a common attachment among the PM capsule, MTL, and PHMM [6, 19]. The MTL was attached at a mean length of 5.9 mm distal to the articular surface of the MTP and extended proximally to the meniscocapsular attachment [6]. Likewise, Smigielski et al. [17] reported that the PM capsule was not attached to the superior edge of the PHMM, forming a wide free posterior femoral recess at the superior aspect of the PHMM. The MTL attached to the inferior aspect of the PHMM was inserted into the posterior proximal MTP. The attachment of the MTL was approximately 7–10 mm distal to the articular surface of the MTP [17]. Peltier et al. [24] reported that the PM capsule was not attached to the posterior aspect of the PHMM. The MTL is firmly anchored at the PHMM; thus, detachment of the ligament could cause rotatory instability of the knee. The relationships of attachments between the PM capsule, MTL, and PHMM are presented in Table 1.

**Table 1:** Location of attachment between the PM capsule, MTL, and PHMM

	Study type	*N*	Meniscocapsular attachment	MTL attachment to the PHMM
Dephillipo et al. [6] Cavaignac et al. [22] Smigielski et al. [17] Peltier et al. [24] Di Francia et al. [28]	Laboratory Laboratory Anatomic Laboratory Laboratory	14 14 NR 4 10	36.4% within the total PHMM height Superior edge of PHMM No attachment (free superior edge of PHMM) No attachment (free superior edge of PHMM) Common junction (PM capsule, MTL, PHMM)	Common junction (PM capsule, MTL, PHMM) Inferior edge of PHMM Inferior edge of PHMM Solidly anchored to PHMM Common junction (PM capsule, MTL, PHMM)

*MTL* meniscotibial ligament, *N* number of cadaveric knees, *NR* not reported, *PHMM* posterior horn of medial meniscus, *PM* posteromedial

Many researchers have studied how the SM tendon and PHMM structures affect the function and motion of the knee joint. It has been suggested that the SM muscle influences the PHMM in the injury mechanism that causes the ramp lesion. Therefore, it is important to understand the relationship between the SM muscle and peripheral attachment of PHMM. Recently, Cavaignac et al. [22] demonstrated the histological relationship between the PHMM and SM tendon and discovered a capsular branch of the semimembranosus (CBSM) attached to the PHMM in all 14 knees, which was composed of collagen fibers but less well organized and dense than the direct tendon fibers. The CBSM extends beyond the PM capsule, superior to the MTL and inferior to the meniscocapsular ligament [22]. An intermediary adipose tissue was newly discovered. It is located posterior to the PHMM, anterior to the CBSM, inferior to the meniscocapsular ligament, and superior to the MTL [22]. Dephillipo et al. [6] reported that the SM tendon is attached to the inferior aspect of the PHMM. On average, the length of this attachment measured 9.2 mm. The attachment site was located at approximately 34.0% of the total axial length of the meniscus, measured from the center of the PHMM’s root [6]. The attachment between SM and PHMM branched from the anterior arm of the SM tendon and was located between the MTL and Posterior oblique ligament (POL) meniscotibial attachments [6]. Kaplan [25] reported three insertions of the SM tendon on the medial aspect of the knee, including insertion into an infraglenoid tubercle and POL, insertion into the coronary ligament and PHMM, and a continuation into the anterior insertion. Laprade et al. [26, 27] found from 20 cadaveric knees that the SM tendon had eight attachments distally, and the direct arm, which bifurcated from the main common tendon, had a broad U-shaped insertion to a bony prominence and additionally attached at the posterior aspect to the MTL. The anterior arm of the SM, which was located medially within the SM bursa and originated from the tibial insertion of the direct arm, deeply extended to the proximal tibial insertion of the superficial medial collateral ligament (MCL). The attachments between SM and PHMM are presented in Table 2.

**Table 2:** Description of the attachment between SM and PHMM

	Study type	*N*	Attachment between SM and PHMM
Dephillipo et al. [6] Cavaignac et al. [22] Kaplan et al. [25] LaPrade et al. [27] Viera et al. [29]	Laboratory Laboratory Anatomic Anatomic Anatomic	14 14 NR NR NR	SM (anterior arm) branched facially to the inferior edge of PHMM Adipose tissue was located between SM and PHMM SM attached to MTL and PHMM SM (direct arm) attached to MTL of PHMM Attached

*MTL* meniscotibial ligament, *N* number of cadaveric knees, *NR* not reported, *PHMM* posterior horn of medial meniscus, *SM* semimembranosus

Various histologic studies of ramp lesions have not yet been sufficiently reported. It has been observed that the outside area adjacent to the periphery of the PHMM consisted of well-vascularized structures indicating the potential for healing if the lesions are adequately preserved or maintained. Di Francia et al. [28] demonstrated that the structure of the meniscosynovial junction was vascularized, and contained nonoriented low-cellularity collagen of moderate density. Cavaignac et al. [22] also reported that the intermediary adipose tissue had a particularly well-vascularized structure. Several studies have reported different histological results of the meniscocapsular and meniscotibial attachments. Some authors explained that linearly organized long fibers were observed equally in both meniscocapsular and meniscotibial attachments and had similar cell densities when PHMM was stained with hematoxylin and eosin [6, 22]. However, Di Francia et al. [28] found that the posterior meniscocapsular attachments were denser than meniscotibial attachments macroscopically. They did not identify the MTL histologically in ten dissected cadaveric knees because the histologic structures of the ligaments, which consists of a dense central collagen band surrounded by loose collagen fibers in the periphery, were absent [28]. They reported that there were loose collagen fibers at 10× and 20× magnifications, which were partially oriented but unparallel, and the dense central collagen band was not discovered microscopically [28].

## Biomechanics

### Function of ramp lesion

Most biomechanical studies have demonstrated a significant increase in anterior tibial translation in knees affected by ramp lesions and ACL tears [13, 30, 31]. In addition, rotational instability, characterized by an increase in internal and external tibial rotation, is also significantly associated with ramp lesions of ACL-deficient knees [13, 24, 31]. This instability could be clinically observed as a high-grade pivot shift [20, 32]. However, it remains uncertain which of the two rotational instabilities plays a primary role in developing a ramp lesion. Ahn et al. [30] conducted a laboratory study of PHMM longitudinal tear in ten human cadavers with ACL-deficient knees in five stages; stage 1, intact knee; stage 2, ACL injury; stage 3, PHMM peripheral longitudinal tear; stage 4, PHMM repair; and stage 5, total medial meniscectomy. The anterior tibial translation was significantly increased at flexion angles 0°, 15°, 30°, and 60° when the PHMM longitudinal tear occurred in an ACL-deficient knee. However, the internal and external tibial rotation did not show significant differences in ACL-deficient knees with peripheral longitudinal tears of the PHMM (stage 3) or with total medial meniscectomy (stage 5) in a combined rotatory load at all flexion angles. Peltier et al. [24] conducted cadaveric studies to evaluate the role of the MTL by dividing it into four stages based on the ramp lesion formation; stage 1, intact knees; stage 2, ACL injury; stage 3, formation of a ramp lesion; stage 4, detachment of the MTL. The results showed a 2.6 mm increase in anterior tibial translation for all flexion angles after forming a ramp lesion with ACL injury (stage 3) compared with the status of ACL injury (stage 2). There was a significant difference in the external tibial rotation between stages 4 and 2 and between stages 4 and 1 for all flexion angles of the knee joint. A cadaveric laboratory study by Dephillipo et al. [13] showed that the anterior tibial translation at 30° and 90° in ACL-deficient knees significantly increased when forming a ramp lesion. The internal and external tibial rotation significantly increased at all flexion angles in ACL-deficient knees with the ramp lesion compared with those with isolated ACL-deficient knees. Stephen et al. [31] also evaluated the rotatory instability of ACL-deficient knees by detaching the meniscocapsular junctions in nine cadaveric knees. The anterior tibial translation at 0–60° and external tibial rotation at 0–40°, 70° showed a significant increase during sectioning of the posterior meniscocapsular junctions in knees with ACL deficiency. The results of the biomechanical study of ramp lesions in ACL-deficient knees are summarized in Table 3.

**Table 3:** Biomechanics of ramp lesions in ACL-deficient knees versus isolated ACL-deficient knees

	*N*	Ramp lesion	AP instability	ER laxity	IR laxity
Ahn et al. [30] Dephillipo et al. [13] Peltier et al. [24] Stephen et al. [31]	10 24 10 9	PHMM peripheral longitudinal tear Detaching PMCA and MTA Detaching MTL Detaching PMCA	(+) 0°, 15°, 30°, 60° (+) 30°, 90° (−) (+) 0°–60°	NR (+) 30°, 90° (+) 0°, 30°, 70°, 90° ( +) 0°–40°,70°	(−) (+) 30°, 90° (+) 0°, 30°, 70°, 90° NR

*AP* anteroposterior, *ER* external rotational, *IR* internal rotational, *MTA* meniscotibial attachment, *MTL* meniscotibial ligament, *N* number of cadaveric knees, *NR* not reported, *PHMM* posterior horn of the medial meniscus, *PMCA* posterior meniscocapsular attachment, (+) Significant increase (*P* < 0.05); (−) No significant increase

Several authors have demonstrated the relationship between ramp lesions and high-grade pivot shift clinically and biomechanically [13, 20, 32]. The function of the medial and PM joint capsule in relation to the pivot shift following an ACL injury is still not fully understood. Dephillipo et al. [13] reported that detaching either posterior meniscocapsular attachment (PMCA) or meniscotibial attachment (MTA) in ACL-deficient knees significantly increases anterior tibial translation and those of internal tibial rotation while stimulating pivot shift test at 15° and 30°. Mouton et al. [20] selected 275 patients with ACL reconstruction and conducted a prospective study on the relationship between ramp lesions and high-graded laxity. Compared with the ACL-injured knees without a ramp lesion, the ACL-injured knees with an isolated ramp lesion had a higher-grade rotatory knee laxity, displaying a grade III pivot shift.

### Injury mechanism of ramp lesion

Among the frequently reported combined injuries reported in cases of acute ACL injury, the most common include ramp lesions of the MM, posterior horn of lateral meniscus (PHLM) root tears, and anterolateral ligament (ALL) injury [33, 34]. While isolated ACL injuries are less common, combined injuries occur frequently [34]. According to cadaver studies, PHLM root tears and ALL injuries can increase rotational knee laxity more than isolated ACL injuries. These injuries may be fixed by restoring rotational stability [35, 36]. Meanwhile, the PHMM is acknowledged as a significant secondary stabilizer in preventing anterior tibial translation [16, 37, 38]. Papageorgiou et al. [16] found from their biomechanical study that the presence of a ramp lesion significantly increases the strain on the reconstructed ACL. It has also been reported that more than 50% of ACL injuries are related to the pivot shift mechanism [39]. Most studies have found that bone contusions to the MTP and MFC in acute ACL injuries are closely associated with ramp lesions [10, 39–41]. Isolated ramp lesions of the MM can occur without an ACL injury. This may be due to the development of ACL longitudinal splits or degeneration [42]. A recent study by Morgan et al. [43] revealed that over 30% of patients with multiligament knee injuries who had an intact ACL were diagnosed with ramp lesions of the MM on MRI. In addition, 66.7% of patients with ramp lesions of the MM also had posterior MTP bone contusions. This implies that surgeons should consider the possibility of ramp lesions in the MM when performing ligament reconstruction, particularly in patients with this bone contusion pattern. Several authors also explained that MCL injuries and medial meniscal tears occurred more frequently as bone contusions with ACL injuries [39, 44]. The progression of bone contusion from the lateral to medial compartment from high-energy transmission at the time of ACL injury is correlated with a high incidence of associated injuries such as MCL injuries, medial meniscal tears, and ramp lesions [39, 44]. Willinger et al. [44] found that 93.7% of all patients with ramp lesions in ACL-deficient knees resulted in superficial MCL injury, and 62.5% of all patients with ramp lesions in ACL-injured knees resulted in deep MCL injury. These concomitant injuries to the deep MCL and superficial MCL with ramp lesions could indicate some specific injury mechanisms. Deep MCL and superficial MCL may also be secondary stabilizers in ACL-deficient knees, similar to the PHMM, when significant anterior tibial and/or external rotatory forces were loaded to the MTP in the ACL-deficient knee [44].

The exact mechanism of a ramp lesion in the PHMM is controversial. One hypothesis is the contrecoup mechanism, suggesting that this mechanism could cause medial compartment bone contusion. Bone contusion refers to the impaction between the posterior MTP and MFC due to a reactive compensatory mechanism, such as varus alignment and internal femoral rotation, during the pivot shift mechanism of ACL injury [10, 39, 40]. To understand the biomechanics of ramp lesions during acute ACL injury, it is necessary to study the actions of the SM muscle and bone contusions associated with PHMM injury [25, 39, 40]. SM contraction occurs suddenly as the traction of the PHMM causes the ramp lesion in the ACL-injured knee while reduction of anterior MTP subluxation was performed [4, 29]. The direct tendon of the SM had an oblique extension to the PM capsule and was also linked to the MTL [25]. Active contraction of the SM could pull the POL and MTL, and Kaplan [25] suggested that posterior displacement of the MM after flexion and internal rotation of the tibia could occur owing to traction applied by the SM muscle. Similarly, Sims and Jacobson [45] proposed that SM tendon exerts a dynamic effect, creating tension within the posterior meniscocapsular complex in PM knee injuries. Vieira et al. [29] dissected the distal tendon of the SM using arthroscopy and reported that, when traction was applied to the distal tendon of the SM, the PHMM was translated posteriorly and the meniscocapsular attachment was stretched.

Another hypothesis for the cause of ramp lesions is the crushing mechanism, in which the PHMM is trapped between the medial femoral condyle (MFC) and MTP with anterior MTP subluxation during an ACL injury accompanied by anterior MTP subluxation [4, 10]. A ramp lesions can occur when high-loading forces are transmitted through the PM capsule during the knee joint valgus stress, internal tibial rotation, and axial compression mechanism to the medial compartment area [21]. The formation of a ramp lesion indicates a connection between contact ACL injuries and MM pathologies [1, 33, 46]. Some authors reported that ramp lesions are more commonly observed in male patients [3, 33], complete ACL injuries [33], and injuries resulting from contact with another person [3, 33]. This mechanism can increase the prevalence of ramp lesions as the incidence of bone contusion at the posterior MTP increases [4, 10].

Ramp lesions in chronic ACL-deficient knee can alter the kinematics of the knee joint by increasing anterior tibial translation and external rotational instability [13, 30, 31]. Previous studies have demonstrated that high-grade pivot shift can also occur in ramp lesions of ACL-injured knees [20]. The crushing mechanism could occur in many cases owing to the increasing laxity of the knee joint in patients with chronic ACL injury. Thaunat et al. [4] reported that complete double longitudinal tears at the meniscocapsular area in the PHMM were likely to develop owing to the crushing mechanism in chronic ACL-deficient knees. In addition, subsequent ramp lesions could develop in chronic ACL-deficient knees owing to repetitive loading forces on the SM muscle [3, 24, 31]. When an anterior loading force was applied to the tibia, the restraining force of the PHMM was found to be significantly greater in the knee with an ACL deficiency compared with that with an intact ACL [21, 38, 47]. Markolf et al. [47] reported that loading anterior and external forces of the tibia produced relatively high forces at the PHMM attachment to stabilize the MM by preventing posterior displacement and impingement between the PHMM rim and MFC. The laxity of the knee with an ACL injury shows a significant increase in anterior tibial translation, leading to high stress at the meniscocapsular junction with the PHMM acting as a mechanical wedge in the MFC [3, 48].

### Risk factors of ramp lesion

Several articles have identified that varus alignment, steep medial meniscal slope, steep medial tibial slope, and deep posterior lateral femoral condyle (LFC) could be risk factors for a ramp lesion [8, 10, 44, 49]. Studies have reported that ACL-deficient knees with ramp lesions had a high predisposition to an increased incidence of MTP bone contusion [10, 40, 41, 44] and the possibility of large varus alignment (> 3$^\circ$) [8, 10]. However, other authors have demonstrated that MTP and MFC bone contusions are not significantly associated with ramp lesions in ACL-injured knees [49, 50]. Dejour et al. [51] demonstrated using a mathematical model when the posterior MTP slope increased by 10$^\circ \mathrm{ or}$ if the anterior tibial translation in ACL-injured knees increased by 6 mm [51]. The tibia is translated anteriorly by the anterior shear force produced by the tibial slope as a reaction to an axial compression load on the knee joint [8, 51]. The steeper the posterior MTP slope, the lower the resistance to the anterior tibial translation [48]. A steep slope also results in more posterior femoral roll-back, which is part of the “contrecoup” mechanism that occurs after an ACL injury. This can lead to impingement of the PHMM by causing it to engage with the MFC [8, 48, 51]. Kim et al. [8] also reported a deep posterior LFC, indicating that a more convex LFC with a less concave lateral tibial plateau (LTP) could be a risk factor for meniscal tears with ACL injury. The deep posterior LFC also increases the length of the ALL complex and decreases the contact area within the joint space of the lateral compartment [8]. This could induce a rotational pivot shift mechanism during ACL injury [8]. The contrecoup mechanism could be revealed due to excessive anterior MTP sliding as a result of a steep posterior MTP slope and high varus alignment (> 3°) [10]. Song et al. [49] demonstrated through a case–control study of a total of 1012 consecutive patients that a high medial meniscal slope (> 3.5°) and chronicity due to delay of ACL reconstruction could be risk factors for ramp lesions with noncontact ACL-injured knees. The biomechanical model suggested that, if the MM thickness decreased as the medial meniscal slope increased, the loading forces on the PHMM would increase, resulting in ramp lesions [49].

However, several articles concluded that a steep posterior MTP slope was not significantly related to ramp lesion incidence in noncontact ACL-injured knees [37, 48, 49]. Hudek et al. [37] explained that a precise comparison of the posterior MTP slope and the medial meniscal slope was difficult because it could not be measured exactly through lateral radiographs. Gender differences could influence the kinematic effect of the steepness of the posterior MTP slope and the risk of ACL injury [37]. Markl et al. [48] also found from 71 acute ACL-injured patients (≤ 3 months) that a higher incidence of meniscal lesions was associated with high posterior MTP and LTP slope (≥ 10°) with respective odds ratios of 2.11 and 3.44. However, it was not a statistically significant difference.

The occurrence of subsequent ramp lesions tends to increase with a delay in ACL reconstruction following injury [3, 5]. Many authors also reported that the time from injury to surgery was a risk factor for ramp lesions in ACL-deficient knees, reporting from 3 to 60 months [3–5, 10, 11, 49]. Subsequent ramp lesion was added from 6.5% (210/3214) to 9.1% (77/868), which is the difference between the incidence of ramp lesions within 3 months and those within 60 months [3, 5], the severity of medial meniscal tears could be more complex than before [11]. Yoo et al. [11] reported that longitudinal or bucket-handle medial meniscal tears were identified on the second preoperative MRI, conducted at an average of 36.8 months after the initial preoperative MRI, with a minimum interval of 6 months. Of the 31 patients, 15 had a longitudinal tear and 2 had a bucket-handle medial meniscal tear on the first preoperative MRI. However, on the second preoperative MRI, there were 19 cases of longitudinal tears and 7 cases of bucket-handle medial meniscal tears, which indicates that 13 knees (42%) had more severe meniscal tears during the second examination. Thaunat et al. [4] demonstrated that the time from injury to surgery of complete tears of ramp lesions was, on average, 16.3 months for type 1, 13.5 months for type 4, and 10.6 months for type 5, which was longer than that of partial tears of ramp lesions, reporting on average 9.5 months for type 2 and 3.3 months for type 3. When ACL reconstruction is delayed, the incidence of complete tears of ramp lesions increases, and the pivot shift grade is also higher.

### Effects of ramp lesion repair

Many authors have concluded that the anterior tibial translation was significantly improved after the ramp lesions of the PHMM were repaired in ACL-reconstructed knees [13, 31]. Several authors also demonstrated that rotational instability after ramp lesion repair would be restored [13, 31]. Ahn et al. [30] reported that repair of the PHMM peripheral longitudinal tear combined with isolated ACL reconstruction without a ramp lesion repair in ACL-deficient knees resulted in a significant decrease in anterior tibial translation at 0°, 15°, 30° and 90° flexion angles compared with isolated ACL reconstruction without a ramp lesion repair in ACL-deficient knees from ten cadaveric knees. Stephen et al. [31] also demonstrated that anterior tibial translation significantly decreased after both ACL reconstruction and repair of PM meniscocapsular junction sectioning at 0–60°. A significantly reduced external tibial rotation was also found from 0° to 40° and 70° to 90° flexion angles [31]. In contrast, resectioning of meniscocapsular junction repair in the ACL reconstructed knee causes a significant increase in anterior tibial translation (at 0°, 10°, 20°, 30°, 40°, and 50°) and external tibial rotation (at 0°, 20°, 30°, 50°, and 60°) [31]. Dephillipo et al. [13] showed from the cadaveric study of 12 matched human knees that repairing ramp lesions in knees that had undergone ACL reconstruction improved the high-grade pivot shift. Repairing meniscocapsular and meniscotibial lesions and ACL reconstruction were needed to improve pivot shift at 15° and 30° flexion angles. However, the internal and external tibial rotation did not improve statistically at flexion angles greater than 30°, although both meniscocapsular and meniscotibial lesions in ACL-deficient knees were repaired with ACL reconstruction. Table 4 presents the results of the biomechanical study conducted after the repair of the ramp lesion.

**Table 4:** Biomechanics after ramp lesion repair

	*N*	Previous status	AP instability	ER laxity	IR laxity
Ahn et al. [30] Dephillipo et al. [13] Stephen et al. [31] Naendrup et al. [52]	10 16 9 9	Ramp lesion in ACL deficiency Ramp lesion in ACLR Detached PMCA in ACLR Intact knee	(+) 0°, 15°, 30°, 90° NR (+) 0°–60° (+) 0°–40°	NR (+) ≤ 30° (+) 0°–40°, 70°–90° (−)	(−) (+) ≤ 30° NR (−)

*ACL* anterior cruciate ligament, *ACLR* anterior cruciate ligament reconstruction, *AP* anteroposterior, *ER* external rotational, *IR* internal rotational, *N* number of cadaveric knees, *PMCA* posterior meniscocapsular attachment, (+) Significant increase (*P* < 0.05); (−) No significant increase

## Epidemiology

The incidence of medial meniscal tears associated with acute ACL injuries has been reported from 23% to 41% [1, 3, 16, 53]. Several authors stated that a longer delay before reconstruction of an ACL deficiency is highly associated with subsequent medial meniscal tears [16, 47, 53]. Julious et al. [53] prospectively evaluated 575 knees of meniscal tears with ACL deficiency and demonstrated that 60.2% of all knees had peripheral posterior horn tears in ACL deficiency, and 40.0% of all knees showed peripheral tears of PHMM in ACL-deficient knees. These peripheral tears of PHMM account for 75.4% of all medial meniscal tears in ACL-deficient knees and 40% of all meniscal tears in ACL-deficient knees [53]. The incidence of ramp lesion in ACL-deficient knees has been reported from 9% to 40% [1, 3, 5, 31, 50]. Liu et al. [3] retrospectively analyzed that ramp lesions were 16.6% in all 868 ACL-injured patients. Recently, Sonnery-cottet et al. [5] reported that the ramp lesions in ACL-deficient patients had an incidence of 23.9% among all 3214 ACL reconstruction patients. Hatayama et al. [50] also evaluated 155 ACL reconstructed knees and found that 46 knees (29.7%) had ramp lesions in all ACL reconstructed knees. The incidence of ramp lesions reported in each article is summarized in Table 5 as a whole.

**Table 5:** Incidence of ramp lesions in different arthroscopic observation studies

	Study type	Incidence (MRI)	Incidence (Arthroscopy)	Anterior view	Transcondylar notch view	PM portal view	Using 70° arthroscope
Kim et al. [10] Liu et al. [3] Hatayama et al. [50] Sonnery-Cottet B et al. [14] Sonnery-Cottet et al. [5] Kim et al. [9] Peltier et al. [23] Dephillipo et al. [41] Dephillipo et al. [56] Okazaki et al. [54] Mouton et al. [20] Willinger et al. [44] Thaunat et al. [4] Seil et al. [1] Tashiro et al. [57]	Retrospective Retrospective Prospective Retrospective Prospective Prospective Prospective Prospective Retrospective Retrospective Prospective Retrospective Retrospective Retrospective Prospective	NR NR 22.6% (35/155) NR NR 21.5% (42/195) 43.6% (17/39) 8.0% (24/301) NR 34.9% (15/43) NR 9% (9/100) NR NR NR	34.5% (95/275) 16.6% (144/868) 29.7% (46/155) 16.6% (50/302) 23.9% (769/3214) 26.6% (50/195) 58.9% (23/39) 16.6% (50/301) 18.6% (158/851) 37.2% (16/43) 21.1% (58/275) 16% (16/100) 15.5% (334/2156) 23.6% (53/224) 9.7% (10/103)	NR NR NR NR NR 9.7% 43.6% NR NR NR NR NR NR NR 0%	NR NR 29.7% 9.6% NR 12.3% 53.8% 16.6% NR 37.2% NR 16% NR NR 9.7%	34.5% 16.6% NR 16.6% 23.9% 26.6% 58.9% NR 18.6% NR 21.1% NR 15.5% 23.6% NR	( +) NR (–) (–) (–) ( +) (–) (–) NR (+) NR (–) NR (+) NR

NR, not reported; PM, posteromedial; (+), Significant increase (*P* < 0.05); (−), No significant increase

## Diagnosis

The most accurate method for preoperative diagnosis of a ramp lesion is through MRI. However, the evaluation during intraoperative arthroscopy through the PM portal is considered the most precise, as some parts of the lesion may not be fully visible through the traditional anterior portal view [9, 14, 23]. The incidence of ramp lesions in ACL-injured patients identified on MRI ranged from 8.0% to 43.6% [9, 23, 41, 44, 50, 54], whereas the incidence of those lesions identified during arthroscopy was higher, ranging from 9.0% to 58.9%. The difference in incidence between MRI and arthroscopy ranged from 2.3% to 15.3%. These lesions are referred to as “hidden lesions” owing to their characteristic location and difficulty in detection [9, 14, 23]. Peltier et al. [23] investigated the relationship between the number of patients with ramp lesions and arthroscopic viewing portals using different approaches, such as transcondylar notch viewing and PM portal viewing. A PM meniscal tear was detected in 17 patients using the traditional anterior portal view. In the transcondylar notch view, four new ramp lesions and three extended known ramp lesions were found. In the PM portal view, six new ramp lesions and five extended known ramp lesions were found [23]. Sonnery-Cottet et al. [14] also performed a systemic arthroscopic exploration of PHMM in a consecutive series of 302 primary ACL reconstructions and reported the “hidden lesions” of the PHMM with ACL injuries. Of the 302 patients, 125 showed a medial meniscal tear, and 50 were in the medial meniscal ramp lesion. Among them, 29 lesions were visualized through arthroscopic exploration of the PM compartment via an anterolateral portal, with the scope positioned deeply into the notch and below the posterior cruciate ligament [14]. Additionally, 21 lesions were discovered by creating an additional PM portal. Kim et al. [9] conducted a consecutive prospective study that evaluated the diagnostic accuracy and suggested steps for the arthroscopic approach to detect ramp lesions in 195 ACL-deficient knees. The sensitivities of detecting the ramp lesions through the traditional anterior portal view and transcondylar notch view using a 30° arthroscope were estimated to be 38% and 48%, respectively. However, the sensitivities of detecting the ramp lesions through the PM portal view and transcondylar notch view using a 70° arthroscope were estimated to be 100% [9]. To accurately diagnose ramp lesions, observation by the PM portal view with a 70° arthroscope should be carefully considered [9, 14]. PM meniscal tears might be difficult to find during ACL reconstruction if surgeons only used the traditional anterior portal view. Therefore, the transcondylar notch view and the PM portal view should be considered to evaluate the meniscal status of PHMM [23].

The diagnostic sensitivity of MRI for detecting ramp lesions associated with ACL injuries has been reported to vary from 69% to 100% [4, 9]. MRI of the knee joint is a relatively accurate diagnostic method for meniscal tears and ligament injuries [11]. Koo et al. [15] performed a systematic review of nine studies from eight articles and reported that the summary sensitivity of the ramp lesions was 0.71 (95% CI, 0.59–0.81) and the summary specificity of the ramp lesions was 0.94 (95% CI, 0.88–0.97) in all preoperative MRI scans of 883 ACL reconstruction patients, which showed moderate sensitivity and high specificity [15].

**Fig. 2:**
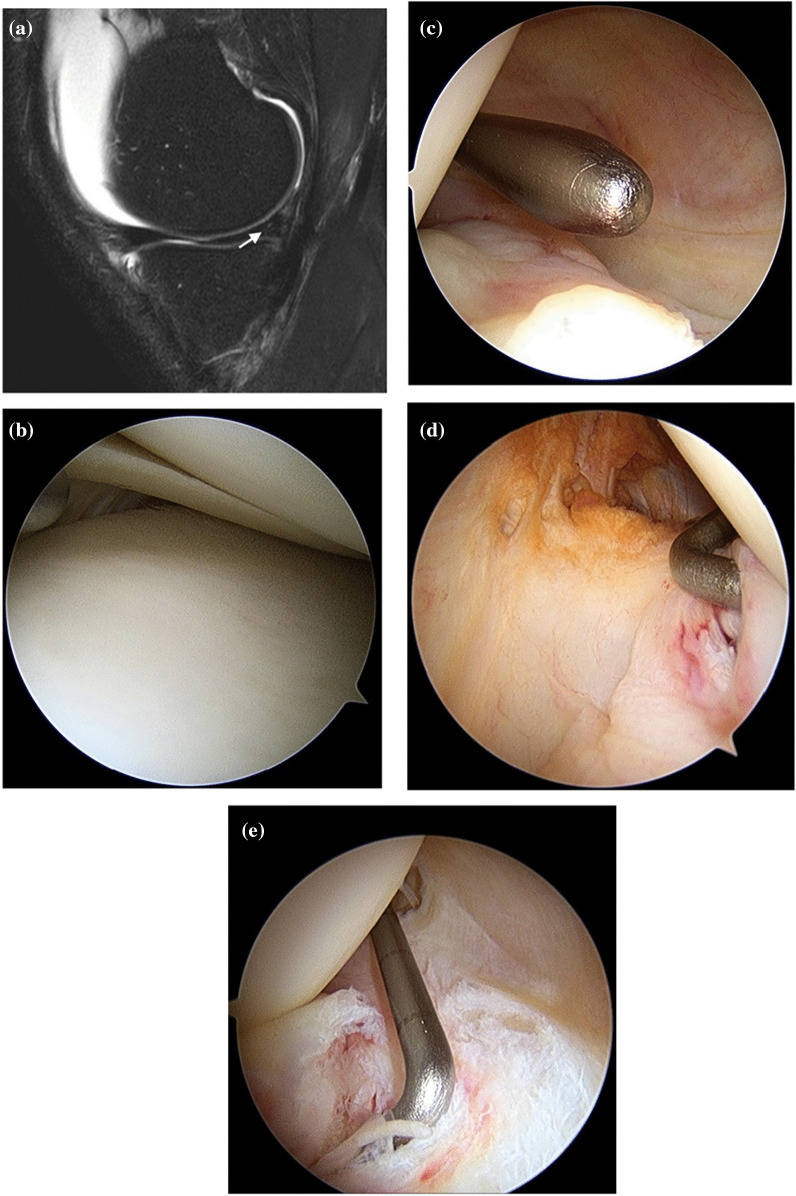
**A** Ramp lesion in an ACL-injured knee on the sagittal plane of preoperative MRI. The signal intensity (white arrow) at PHMM was high, but it was uncertain whether there was a definite tear. **B** Anterior view using 30° arthroscopy. The medial meniscal tears were not confirmed during the anterior view examination using 30° arthroscopy and probing. **C** Transcondylar notch view using 30° arthroscopy. It was uncertain whether there was a definite tear in the peripheral area of the PHMM. **D** Posteromedial (PM) portal view using 70° arthroscopy. The tear was confirmed by probing through the transcondylar notch with a posteromedial portal view using 70° arthroscopy. **E** Transcondylar notch view using 70° arthroscopy. Ramp lesions could be easily identified

High-resolution MRI and patient knee flexion position contributed to increased sensitivity of the ramp lesions, in particular in up to 84% of patients [15]. However, some articles reported no significant difference between 1.5-T and 3-T MRI [50]. Ramp lesions are difficult to find with a fully extended knee in ACL deficiency because this position could create a narrow space between the PHMM and the PM capsule of the knee joint [3]. Yeo et al. [55] classified the MRI features of ramp lesions into six categories: complete fluid filling between the PM capsule and PHMM, posterior edema affecting the PM capsule, posterior marginal irregularity of the PHMM, perimeniscal fluid sign, corner notch sign, and PHMM vertical tear. Complete fluid filling between the PM capsule and PHMM and posterior marginal irregularity of the PHMM were the most sensitive findings to detect ramp lesions on MRI [55]. A recent survey was conducted, which showed that only 14% of knee surgeons were likely to confirm the PM meniscocapsular junction area routinely to evaluate ramp lesions in ACL-deficient knees [15]. Therefore, it is necessary to check the “hidden lesion” during ACL reconstruction even if a ramp lesion is not discovered on preoperative MRI [9, 14, 15]. Figure 2A and B show a ramp lesion and an ACL rupture, respectively, on the sagittal plane of preoperative MRI. Figure 2C, D, and E display intraoperative arthroscopic views from different portals. Table 5 presents the incidence of ramp lesions in ACL-injured patients as observed from the anterior view, transcondylar notch view, PM portal view, and using 70° arthroscopy.

## Study limitations

This review has several limitations. The first is that not all articles on ramp lesions were included in this review. There was a potential existence of unpublished studies that may have negative or inconclusive findings, which were not included in the analysis. Second, further studies are required to investigate peripheral anatomical attachments of PHMM and histological evaluations of the lesion. More histological studies focusing on the attachments of the PHMM are needed to evaluate the injury mechanism of ramp lesions associated with ACL injury and to analyze the reasons for the increased incidence of posterior medial meniscal lesions in ACL-deficient knees. The third limitation is that, although there have been many ongoing studies regarding anatomical attachments, risk factors of ramp lesions, injury mechanisms of the lesions, and diagnosis of the lesions, they have not yet been clearly demonstrated. Detailed analysis of risk factors is required to understand the anatomy and biomechanics of ramp lesions associated with ACL deficiency. Fourth, the treatment of ramp lesions is not included in this review.

## Conclusions

Ramp lesions are commonly observed in patients with ACL injuries. It has been suggested that the superior and inferior edges of the PHMM are connected to the meniscocapsular ligament and MTL. By identifying specific common junction structures between PHMM, MTL, PM capsule, and the SM muscle, there is a high possibility that the ramp lesion may be influenced by the SM muscle. The injury mechanism of ramp lesion is believed to occur as a contrecoup injury due to compensatory reactions from high-energy injury of the ACL. The PHMM is a secondary stabilizer of the laxity of the knee joint. It is known that ramp lesions can lead to AP instability. According to recent studies, more reports suggest that concurrent injuries such as LM root tear and ALL injury may contribute to rotational instability and ramp lesions. The risk factors of ramp lesions include varus alignment, steep medial meniscal slope, steep medial tibial slope, and deep posterior LFC. Chronicity is another risk factor that can increase the incidence of subsequent ramp lesions in ACL-deficient knees and contribute to the development of ramp lesions. Although a ramp lesion was not detected on preoperative MRI before ACL reconstruction, careful evaluation of the lesion is needed by PM portal and transcondylar notch view using 70° arthroscopy. Accurate diagnosis of ramp lesions through MRI and arthroscopy is considered to significantly affect the patient’s prognosis.

## Data Availability

Not applicable.
